# Granuloma Multiforme: A Rare Granulomatous Disease

**DOI:** 10.1155/2019/5485034

**Published:** 2019-10-07

**Authors:** Yogesh Poudyal, Anita Shah

**Affiliations:** ^1^Sarnath Skin Centre, Shanti Path, Bhairahawa 32900, Nepal; ^2^Department of Pathology, Universal College of Medical Sciences-Teaching Hospital, Ranigaon, Bhairahawa 32900, Nepal

## Abstract

Granuloma Multiforme (GM) is a reactive skin disorder with annular lesion and focal necrobiosis. It was first described in Nigeria (Africa), since than it was mostly described from various regions of Africa where leprosy was endemic. Outside Africa, this disease is rarely reported. Till 2016 only five cases were reported from India. This is the second case report from Nepal. A fifty-nine-year-old female, farmer by occupation, presented with annular and polycyclic plaques with elevated beaded border for one year. It was distributed over upper trunk, lower back, and arms, and was mildly pruritic. Histopathologically, loss of elastic fibres at focal area of upper dermis, with elastic fibres engulfed histiocytes were noted. GM was mostly noted from leprosy endemic areas of Africa. Clinically, it is very difficult to distinguish from granuloma annulare. This report adds the number of GM occurrence in Asia especially from area where leprosy is still prevalent. This report also emphasizes in resource poor settings where most diagnosis is made clinically, the granuloma annulare should be looked for very carefully in order not to miss GM.

## 1. Introduction

Granuloma Multiforme is a reactive skin disorder of unknown aetiology, characterized clinically by confluent annular lesions and histologically by focal necrobiosis and histiocytic granuloma [1]. It was first described by D. L. Leiker, which was endemic in Mkar, Nigeria. It was identified when lesion resembling leprosy was not responding to sulphone treatment, as this disease was misdiagnosed and treated as leprosy. Later this disease was noticed to be of high prevalence in South Eastern Nigeria, West Kenya, and Isle of Sumba in Indonesia [2]. Very few reports were found from other parts of the world. Even from India, till 2016 only five cases were reported [3].

## 2. Case Presentation

A fifty-nine-year-old female, from Western Terai region of Nepal, presented with slightly itchy lesion on trunk and arm for one year. The first lesion was noted on chest, then gradually new lesions were seen on the arm and lower back. It started as skin color small palpable lesion that gradually increased in size giving ring shape. There was no history of other disease and drug intake. She was a farmer by occupation.

**Figure 1:**
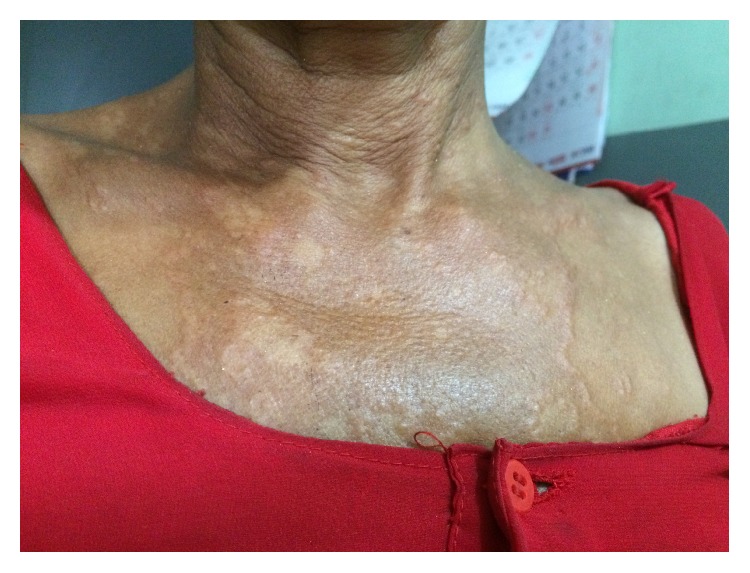
Annular and polycyclic plaques on chest.

**Figure 2:**
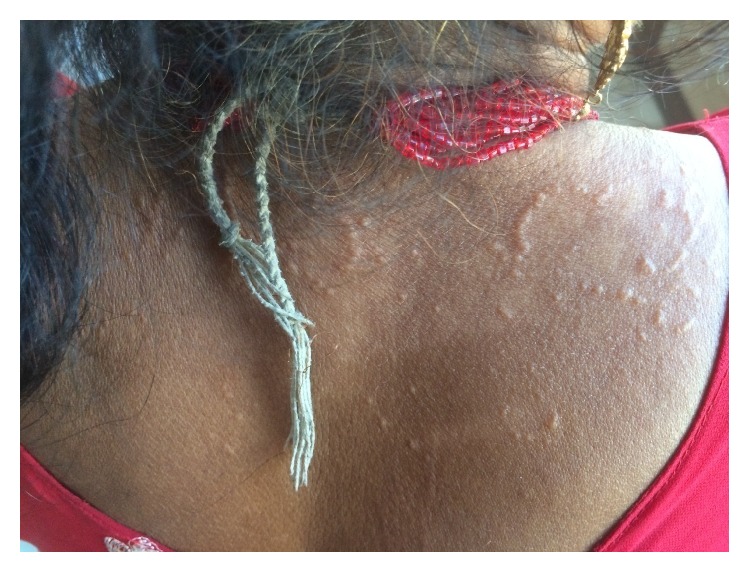
Annular and polycyclic plaques with beaded well defined border on upper back.

**Figure 3:**
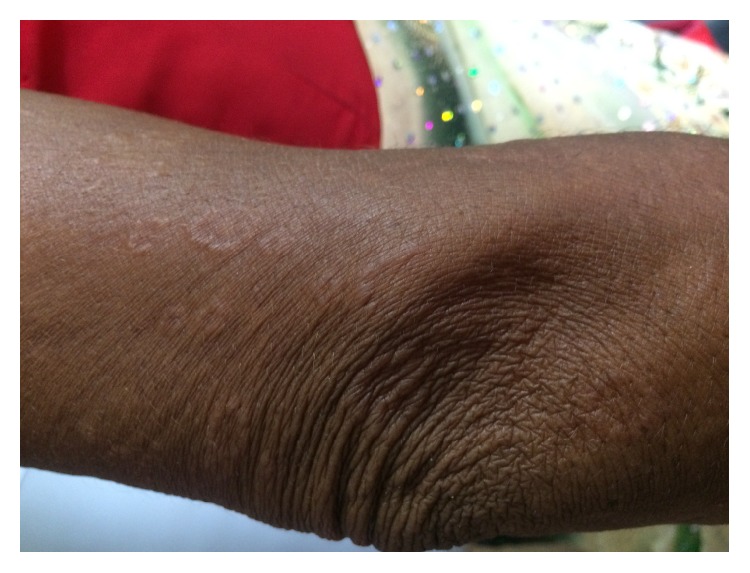
Annular plaques with well defined border on arm.

On examination, there were multiple skin color and slightly erythematous annular and polycyclic plaques with elevated, beaded border with central clearing. Lesions were distributed over chest, forearm, upper, and lower back with size ranging from 2 cm to 10 cm in diameter (Figures 1–3). The sensation was intact and peripheral nerves were not thickened.

The clinical possibilities considered were granuloma annulare, leprosy, annular sarcoid, and actinic granuloma.

Complete haemogram, renal and liver function test, blood glucose level, and routine urine examination were within normal range.

**Figure 4:**
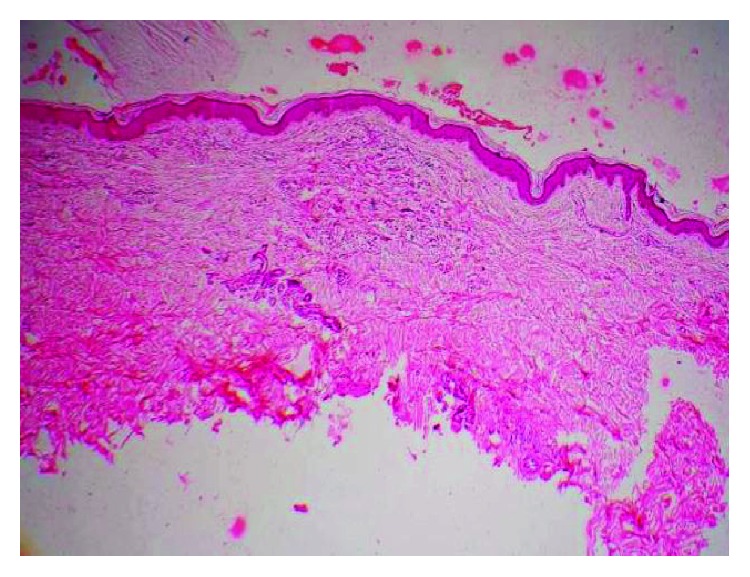
Multinucleated histiocytes abutting connective tissue with elastotic degeneration (H&E stain with 4x magnification).

**Figure 5:**
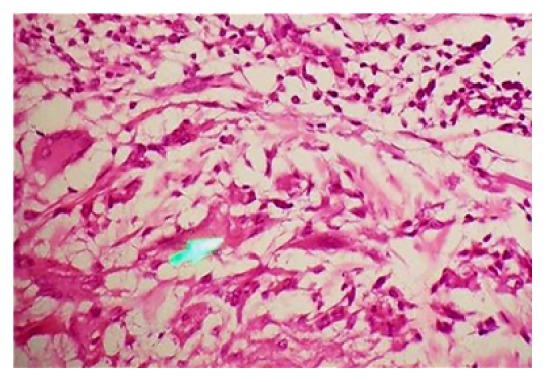
Multinucleated giant cell with ingested elastic fibres (arrowhead); (H&E stain 40x magnification).

The elliptical incisional biopsy was taken from the margin of lesion and sent for histopathological examination. It showed keratinized stratified squamous epithelium with normal maturation. There was loss of elastic fibres at focal area of upper dermis (Figure 4). The periphery of the lesion showed many multinucleated giant cell and histiocyte along with few lymphocyte and plasma cell. Engulfed elastic fibres by multinucleated giant cells were also noted (Figure 5).

The patient was prescribed topical steroid, but showed no improvement at follow up after six weeks.

## 3. Discussion

Granuloma Multiforme is found among adult over the age group of 40 years with the predilection for the female sex. The sun exposed sites of the upper trunk and arms are predominantly affected. The initial lesions are usually papules, which soon evolve to form annular and polycyclic lesions with papular or nodular edge. The lesion tends to last for months or years. It is pruritic and irritating, especially when new lesions are forming [1]. Scattered case reports of GM were seen in recent times from Congo [4], India [5], Tunisia [6].

Since the time first described, its important differential diagnosis has been leprosy. The absence of loss of sensation and normal peripheral nerves will easily rule out leprosy. Granuloma Annulare (GA) sometime without histopathological support can be very difficult to differentiate. GA has smaller lesion and is seen in children. The presence of palisading granuloma differentiates it from GM. Annular sarcoid will show naked granuloma histologically and infiltrated reddish brown plaque clinically. Actinic granuloma or Annular elastolytic giant cell granuloma can be differentiated by its asymptomatic nature and absence of necrobiosis in histology. Some authors believe Actinic granuloma and GM are the same entity.

The exact aetiology of this disease is not known. In past there were reports even suspecting droppings of bat, leaves as the aetiological factors but none were confirmed [2]. It is suggested that GM is due to cumulative damage to collagen by sunlight induced by chemical or biological agent in environment and potentiated by immunologic factor [7]. Like previous reports [3, 5, 7–9], our case too showed female gender with age above forty. The propensity in females is difficult to explain but we should remember that other photodermatosis like Lupus erythematosus also shows a strong predominance in women [7]. The occupation as farmer and residence at Western Terai region, where in summer temperature rises above 40ºC, shows the possibility of photoexposure for a long time.

There is no effective therapy for this condition, but recent report has shown some response with dapsone [5].

This is the second reported case from Nepal [10]. Like most of the previous reports, we have a female above forty. There are very few reported cases for this disease, this can be probably due to misdiagnosis as there is striking similarity of this case with Granuloma Annulare. Due to rarity of occurrence of GM, it might have been over looked in most instances. High degree of suspicion is required, while dealing with the granulomatous disorder of skin especially Granuloma Annulare. With this report and few others from India, it can be emphasized that this disease is not only confined to Africa, but also at Asian Subcontinent.
